# Steviol glycosides as an alternative osmotic agent for peritoneal dialysis fluid

**DOI:** 10.3389/fphar.2022.868374

**Published:** 2022-08-16

**Authors:** Valeria Kopytina, Lucía Pascual-Antón, Nora Toggweiler, Eva-María Arriero-País, Lisa Strahl, Patricia Albar-Vizcaíno, David Sucunza, Juan J. Vaquero, Sonja Steppan, Dorothea Piecha, Manuel López-Cabrera, Guadalupe-Tirma González-Mateo

**Affiliations:** ^1^ Department of Immunology, Molecular Biology Research Center Severo Ochoa (CBMSO), Spanish National Research Council (CSIC), Madrid, Spain; ^2^ Fresenius Medical Care Deutschland GmbH, Frankfurter, St. Wendel, Germany; ^3^ Department of Nephrology, IdiPAZ Research Institute, La Paz University Hospital, Madrid, Spain; ^4^ Department of Organic and Inorganic Chemistry, Faculty of Pharmacy, University of Alcalá (IRYCIS), Madrid, Spain; ^5^ Fresenius Medical Care Deutschland GmbH, St. Wendel, Germany

**Keywords:** biocompatibility, dialysis solutions, fibrosis, osmotic agents, peritoneal dialysis (PD), chronic inflammation

## Abstract

**Background:** Peritoneal dialysis (PD) is a renal replacement technique that requires repeated exposure of the peritoneum to hyperosmolar PD fluids (PDFs). Unfortunately, it promotes alterations of the peritoneal membrane (PM) that affects its functionality, including mesothelial-mesenchymal transition (MMT) of mesothelial cells (MCs), inflammation, angiogenesis, and fibrosis. Glucose is the most used osmotic agent, but it is known to be at least partially responsible, together with its degradation products (GDP), for those changes. Therefore, there is a need for more biocompatible osmotic agents to better maintain the PM. Herein we evaluated the biocompatibility of Steviol glycosides (SG)-based fluids.

**Methods:** The ultrafiltration and transport capacities of SG-containing and glucose-based fluids were analyzed using artificial membranes and an *in vivo* mouse model, respectively. To investigate the biocompatibility of the fluids, Met-5A and human omental peritoneal MCs (HOMCs) were exposed *in vitro* to different types of glucose-based PDFs (conventional 4.25% glucose solution with high-GDP level and biocompatible 2.3% glucose solution with low-GDP level), SG-based fluids or treated with TGF-β1. Mice submitted to surgery of intraperitoneal catheter insertion were treated for 40 days with SG- or glucose-based fluids. Peritoneal tissues were collected to determine thickness, MMT, angiogenesis, as well as peritoneal washings to analyze inflammation.

**Results:** Dialysis membrane experiments demonstrated that SG-based fluids at 1.5%, 1%, and 0.75% had a similar trend in weight gain, based on curve slope, as glucose-based fluids. Analyzing transport capacity *in vivo*, 1% and 0.75% SG-based fluid-exposed nephrectomized mice extracted a similar amount of urea as the glucose 2.3% group. *In vitro*, PDF with high-glucose (4.25%) and high-GDP content induced mesenchymal markers and angiogenic factors (Snail1, Fibronectin, VEGF-A, FGF-2) and downregulates the epithelial marker E-Cadherin. In contrast, exposition to low-glucose-based fluids with low-GDP content or SG-based fluids showed higher viability and had less MMT. *In vivo,* SG-based fluids preserved MC monolayer, induced less PM thickness, angiogenesis, leukocyte infiltration, inflammatory cytokines release, and MMT compared with glucose-based fluids.

**Conclusion:** SG showed better biocompatibility as an osmotic agent than glucose *in vitro* and *in vivo*, therefore, it could alternatively substitute glucose in PDF.

## Introduction

Peritoneal dialysis (PD) is a well-established kidney replacement therapy that allows the elimination of toxic metabolic products and excess water from the body, through a process known as ultrafiltration (UF). However, continuous exposure to PD fluids (PDFs) triggers a series of processes associated with the appearance of morphological and functional alterations in the peritoneal membrane (PM), leading to the development of fibrosis, angiogenesis, hyalinizing vasculopathy, and a deterioration of the membrane UF capacity (Krediet, 1999). These alterations are attributed to the lack of biocompatibility of PDFs, which generally use glucose as an osmotic agent. In addition, heat sterilization of glucose-based PDFs generates glucose degradation products (GDP) (Welten et al., 2003), which in turn raises the formation of advanced glycation end-products (AGEs). AGEs, together with GDP, promote the synthesis of cytokines related to inflammation, fibrosis, vascular alterations, and angiogenesis, driving UF failure (Qayyum et al., 2015).

Moreover, PDFs cause damage to the mesothelial cells (MCs) monolayer that lines the whole PM and is in direct contact with the PDFs. As a consequence of these damages, MCs undergo a process called mesothelial-mesenchymal transition (MMT). MMT is a normal tissue repair process by which cells acquire the ability to migrate, invade the submesothelial stroma and synthesize large amounts of extracellular matrix as well as inflammatory and angiogenic factors (Yáñez-Mó et al., 2003; López-Cabrera, 2014). PDF exposure leads to tissue damage and consequently generates inflammation, inducing pathological MMT that plays a key role in the peritoneal injury. Bio-incompatible PDFs also alter the peritoneal leukocytes population, which constitutes the first line of defense against peritoneal infection (McIntyre, 2007; García-López et al., 2012). The repeated metabolic and biomechanical damages arising from serial PDF exposures lead to smoldering inflammation and reduced host defense in the peritoneal cavity (Jörres et al., 1992; Brulez et al., 1999; Devuyst et al., 2002; Mackenzie et al., 2003; Schilte et al., 2009). The interplay of PDF cytotoxicity and intermittent bacterial infections contribute to clinical complications of PD therapy, such as UF failure and peritonitis (Davies et al., 1998).

During the last decades, new and more biocompatible PDFs have been developed to correct the problems associated with PD by using either different osmotic agents such as icodextrin or amino acids, or other sterilization techniques to avoid the generation of GDP or more physiological pH. However, a definitive solution has not been found yet. Glucose is still the most widely used agent since other commercialized substitutes (icodextrin and aminoacids) can only be used in one exchange per day, so it is mandatory to use glucose in the rest of the exchanges. The constant use of a high amount of glucose not only causes local problems in the peritoneum but also systemic alterations, such as diabetes, dyslipidemia, hydrocarbon intolerance (Delarue and Maingourd, 2001), and cardiovascular disease (Balafa and Krediet, 2012). Meta-analyses revealed no significant improvement of newer varieties of biocompatible PDFs on peritonitis rate or PM function (Cho et al., 2013, 2014). Therefore, the greatest challenge since the development of PD as a treatment for chronic kidney disease (CKD) is to find a definitive alternative to glucose.

*Stevia rebaudiana* is a plant that is used as a sweetener (Goyal et al., 2010). The leaves of *stevia rebaudiana* contain Steviol glycosides (SG) which are active compounds responsible for their sweet taste. Many plant-based glycosides are used as medicines. Beyond sweetening value, SG have therapeutic effects against cancer, inflammation, cystic fibrosis, obesity, and dental caries (López et al., 2016; Momtazi-Borojeni et al., 2017; Ramos-Tovar et al., 2018; Casas-Grajales et al., 2019). Another aspect that makes it even more attractive is that it acts as an antioxidant (Bender et al., 2015), anti-hypertensive (Hsieh et al., 2003), anti-diabetic, improving hydrocarbon resistance (Mohd-Radzman et al., 2013; Periche et al., 2015), and protects against kidney damage (Ozbayer et al., 2011; Shivanna et al., 2013). Hypertension, hydrocarbon intolerance, and a pro-oxidative environment frequently accompany central obesity, being cardiovascular risk factors commonly found in uremia (a syndrome caused by the accumulation of toxic products in the blood that the kidneys are not able to eliminate) (Podkowińska and Formanowicz, 2020), therefore SG properties could help to control these illnesses.

An optimal osmotic agent for PD should have some specific characteristics, such as a molecular weight (MW) that permits using it in several exchanges per day, an osmotic and UF capacity, no absorption, no interference with tissue repair ability nor immunocompetence, and no toxicity (Khanna et al., 1986). This is important as part of it can be absorbed or degraded, thus constituting an additional problem (Lee et al., 2005). Moreover, it has been determined that an ideal osmotic agent should weigh around 1 kDa (Rippe and CarlssOn, 1996). According to this background, we selected a pure *stevia* extract composed of SG (≥ 95%), which contains Rebaudioside A as the main compound (62.6%) and Stevioside (21.3%) to be tested as an osmotic agent for PDFs. The components of the *stevia* extract do not contain glucose and have MW ranging from 950 to 1,050 Da.

## Materials and methods

### Peritoneal dialysis fluids

A mixture of SG (≥95%) that contains Rebaudioside A (62.6%) and Stevioside (21.3%) was bought from AgriStevia (SG95RA60, Spain). The total composition and theoretical osmolarity of SG extract are indicated in the Supplementary Figure SA,B. SG solutions were prepared at different concentrations (0.75%: pH = 6.29; 1%: pH = 6.24, 1.5%: pH = 6.17) in a lactate buffer that contains: 1.25 mmol/L calcium, 134 mmol/L sodium, 0.5 mmol/L magnesium, 100.5 mmol/L chloride, 25 mmol/L lactate. SG solutions have been heat sterilized at 121°C for 1 h for *in vitro* and *in vivo* experiments. PDFs containing 1.5%, 2.3%, and 4.25% dextrose (glucose monohydrate, Mass Weight 198.20 Da) are commercially available and labeled as such in North America. The true anhydrous dextrose or glucose concentrations (Mass Weight 180.15 Da) in these solutions are 1.36%, 2.27%, and 3.86%, respectively, and this is how they are typically labeled in Europe. We used American nomenclature in this work (Daugirdas et al., 2015). A commercial standard PDF with 4.25% of glucose, pH = 5.5, and a high concentration of GDP (Stay Safe^®^; Fresenius Medical Care) was used as a positive control (named SS 4.25%), and a more biocompatible PDF with 2.3% glucose, pH = 7.0, and low-GDP concentration (Balance^®^, Fresenius Medical Care) (named Bl 2.3%) was employed to compare its effects *in vitro* and *in vivo* with SG.

### Osmotic capacity measurements

Glucose-based PDFs at 1.5%, 2.3%, and 4.25% concentrations have been used as commercial dialysis solutions to compare their osmotic capacity with SG solutions. The solutions contain glucose solved in different concentrations in a lactate matrix with the same composition as in commercial SS 4.25% and Bl 2.3% PDFs. Glucose-based solutions have been heat sterilized at 121°C for 1 h. SG-based fluids were used at 1.5%, 1%, and 0.75% concentrations. pH-value of all test solutions was adjusted to 6.4 ± 0.2 using hydrochloride acid or sodium hydroxide before performing the experiments.

Osmotic capacity experiments were performed using dialysis membranes (ZelluTrans/Roth V-series) with a molecular weight cutoff 25 kDa purchased from Carl Roth. Dialysis tubing length of 8 cm was used in all experiments. The filling volume of dialysis tubes at the beginning of the experiments was 10 ml, closed by two clamps, each system with a sinker and swimmer. Dialysis tubing bags were placed in a beaker filled with 1,100 ± 0.1 g of lactate matrix which is tempered to 37°C and the weight of each bag was measured to determine the mass change all 30 min over a period of 24 h. The mass increase was calculated for each dwelling time interval. Experiments were carried out 3-times, and the mean value with the respective standard deviation was calculated. Determination of the concentration of the osmotic agent was made by measuring the refraction index (Refractometer DR 6300-T, KRÜSS Optronic). A calibration curve was prepared for each substance, and the test solution was measured after 24 h’ test.

The reflection coefficient of the SG mixture was calculated using the relation between reflection coefficients (*σ*) of low molecular weight solutes (urea, glycerol, glucose, sucrose) as well as insulin, icodextrin, and albumin and their molecular weights.

### Isolation, culture, and treatments of human omental peritoneal MCs

HOMCs were obtained from omental samples taken from patients undergoing elective abdominal surgery as described previously (Stylianou et al., 1990; Aroeira et al., 2005). Cells were cultured in Earle’s M199 medium (Biological Industries, Kibbutz Beit Haemek, Israel) supplemented with 20% fetal bovine serum (FBS; Thermo Scientific, Cramlington, United Kingdom), 50 U/ml penicillin, 50 μg/ml streptomycin (PPA Laboratories GmbH, Pasching, Austria), 2% HEPES 1 M (Lonza™ BioWhittaker™), 2% Biogro-2 (Biological Industries). These cells were used and remained stable for one to two passages.

HOMCs were incubated with glucose-based PDFs (SS 4.25%, or Bl 2.3%, Fresenius Medical Care) diluted 1:1 with a culture medium for 48 h. In addition, HOMCs were incubated with SG 1% or 0.75% in the same conditions. HOMCs were also treated with recombinant human TGF-β1 (1 ng/ml) (R&D Systems Inc., Minneapolis, MN, United States) to induce MMT *in vitro* (Aroeira et al., 2005). The control (CTRL) group was incubated with a lactate buffer 1:1 with a culture medium. Each experiment was carried out in duplicate, and at least five experiments were performed.

### Culture of Met-5A and viability assay

The stable tetrazolium salt MTT is converted by active mitochondrial dehydrogenases of living cells to MTT Formazan. Therefore, the amount of formazan dye formed directly correlates to the number of metabolically active cells in the culture.

Met-5A cells were grown in a 96-well tissue culture plate in Earle’s M199 medium (Biological Industries, Kibbutz Beit Haemek, Israel) supplemented with 10% fetal bovine serum (FBS; Thermo Scientific, Cramlington, United Kingdom), 50 U/ml penicillin, 50 μg/ml streptomycin (PPA Laboratories GmbH, Pasching, Austria), 2% HEPES 1 M (Lonza™ BioWhittaker™), 2% Biogro-2 (Biological Industries). The cells were incubated with glucose-based PDFs (SS 4.25%, or Bl 2.3%, Fresenius Medical Care) and SG solutions 1% or 0.75%, as well as with TGF-beta (1 ng/ml) and DMSO 5% as controls, diluted 1:1 with a culture medium for 96 h. The cells were incubated subsequently with the MTT reagent (Invitrogen, reference M6494) at 0.5 mg/ml for 4 h. Then, DMSO was added to solubilize the formazan dye formed and it was quantitated with a scanning multi-well spectrophotometer at 570 nm (ClarioStar Plus reader). The measured absorbance directly correlates to the number of viable cells.

### Real-time qPCR

Cell lysis and RNA extraction were done using TRI Reagent^®^ Solution (Ambion, Austin, TX) according to the manufacturer’s instructions. cDNA for RT-qPCR was generated from 1 μg total RNA using the High Capacity cDNA Reverse Transcription Kit (Applied Biosystems, Foster City, CA, United States). Quantitative PCR was carried out on a LightCycler 480 using a SYBR Green labeling kit (Roche, Mannheim, Germany) and specific primers sets for human histone H3, Snail1, Fibronectin, VEGF-A and E-Cadherin. The data were analyzed by using the comparative Ct method (ΔΔCt). The x-fold change in RNA expression was quantified relative to control samples from the same experiment. Samples were normalized with respect to the value obtained for H3.

### FGF-2 and VEGF-A detection in supernatants

The supernatants were collected and stored at −80°C. The secreted Fibroblast Growth Factor 2 (FGF-2) and VEGF-A by HOMCs were determined using a simplex kit (EPX01A-12074-901, EPX01A-10277-901, Invitrogen) by Luminex 100 technology.

### Animals

Female C57BL/6JRccHsd mice, from 18 weeks of age, were purchased from ENVIGO (Barcelona, Spain) and maintained in conventional conditions at the CBMSO animal facilities. All animal procedures were approved by the ethics committee of the CSIC, authorized by the Comunidad Autónoma de Madrid according to RD 53/2013, with affiliation number PROEX 273/19, and conducted in accordance with the institutional guidelines that comply with the Directive of the European Parliament and of the Council on the Protection of Animals Used for Scientific Purposes.

### Urea removal capacity and surgery

For urea removal capacity analysis, mice were submitted for surgery to induce uremia (González-Mateo et al., 2018). Briefly, 5/6 nephrectomy was performed under isoflurane anesthesia (4% for induction and 2%–3% for maintenance); 0.05–0.1 mg/kg buprenorphine (Temgesic) was injected intramuscularly 15–30 min preoperatively, and eye drops were given. The animal was shaved in the abdomen, and it was placed on a heating pad. An incision of approximately 0.5 cm in the skin was performed, on the right side, close to the ribs, to have direct access to the right kidney. A small incision was opened in the muscle to take the right kidney out of the peritoneum, removing the capsule and the adrenal gland. At this point, the kidney could be easily positioned on top of the peritoneum and was placed on a wound pad. A total ligation with insoluble suture was applied that included the kidney vein, artery, and urethra. After the ligation, the right kidney was totally removed from the body. After 1 week of recovery, the left kidney was also removed from the abdominal cavity following the same procedure and released from the capsule. The anterior one-third and posterior one-third parts of the kidney were impaired by using a monopolar electric blade. The remaining functional one-third of the left kidney is placed back into its original position in the abdominal cavity. To allow the mice to fully recover, transport analyses were performed 2 weeks after surgery.

The mice were euthanized 10, 40, and 60 min after receiving 2 ml of each tested fluid (Bl 2.3%, SG 1%, and SG 0.75%, *n* = 4 or 5 per group), and the total peritoneal volume was drained and urea concentration was measured. To confirm uremia, a volume of 200–400 μl blood was drawn via facial vein puncture 2 days before the surgery and at the end point, analyzing serum samples for urea levels (Ab83362; Abcam).

### PD fluid exposure and surgery

Catheter implantation was performed as previously described (Aroeira et al., 2009; González-Mateo et al., 2018). The control group received physiologic saline (605,137.5, Grifols, *n* = 5 per group). The glucose-based PDF groups received a commercial solution with high glucose and high-GDP concentration or low glucose and low-GDP concentration (PDF-4.25% glucose; pH 5.5; Stay Safe^®^; and PDF-2.3% glucose, pH 7.0; Balance^®^, respectively, from Fresenius Medical Care) (*n* = 6 per group). The SG groups received SG at 1% and 0.75% concentrations diluted a lactate buffer (*n* = 6 per group). All mice received 2 ml of the solution corresponding to their treatment group, twice a day through the catheter, for 40 days. Before starting the 40 days-treatment, animals were injected with increasing concentrations of the corresponding fluid, starting at a 5 times-diluted concentration and increasing proportionally every 3 days for 12 days until the final concentration was reached for each group. This methodology was designed in order to avoid possible undesirable reactions due to a high-concentrated initial exposition, as we have previously observed that animals need to gradually adapt to SG intraperitoneal (i.p.) exposition.

### Cytokines detection in peritoneal washings

Peritoneal washings were collected at the end of the experiment, stored at −80°C, and analyzed by Luminex 100 technology using simplex kits for IL-6 (EPX01A-20603-901, Invitrogen), IL-22 (EPX01A-26022-901, Invitrogen), IL-1β (EPX01A-26002-901, Invitrogen), TNF-α (EPX01A-20607-90, Invitrogen), IL-17 (EPX01A-26001-901, Invitrogen).

### FACS analyses

Cell suspensions obtained from peritoneal washing were counted with a Scepter handheld automatic cell counter (Millipore) and stained with fluorochrome-conjugated mouse-specific antibodies against CD3, CD4, CD8α, CD11b, Ly6G, F4/80 (BD Biosciences Pharmingen, San Diego, CA, United States) following manufacturer’s instructions. Samples were analyzed in a BD FACS Canto II (BD Biosciences, San Jose, CA) flow cytometer, and data analyses were performed using FlowJo software.

### Histological analyses

Parietal peritoneal biopsies were collected from the opposite side of the catheter installation. For thickness analyses, biopsies were fixed in Bouin solution, embedded in paraffin, cut into 5 μm sections, and stained with Trichrome Stain (Masson) kit (HT15, Sigma-Aldrich, United States). PM thickness of submesothelial tissue was determined by blinded microscope analysis using a metric ocular. The peritoneal thickness in each mouse was calculated by the median of measurements taken every 50 μm from one extreme to the other of the biopsy. The result was used to calculate the group thickness.

For immunofluorescence staining, biopsies of mice were frozen in optimal cutting temperature (OCT) compound and cut into 5 μm sections. To identify the mesothelial cells, we used a mouse anti-cytokeratin (CK) 8/18 (clone 5D3; Novocastra, Newcastle, United Kingdom), which was stained with anti-IgG1–specific Zenon Fab Fragments (Zenon Alexa Fluor 555, Invitrogen) according to the manufacturer’s instructions. The CD45^+^ cells were identified using a rat anti-mouse CD45^+^ (Clone 16A; Biotin Rat Anti-Mouse CD45RB, BD Biosciences) and stained with goat anti-rat antibody (Alexa Fluor 488, A11006, Invitrogen). Fibroblastoid cells were marked with rabbit anti-mouse FSP-1 (Polyclonal Rabbit Anti-Human S100A4, Dako, Denmark) and stained with goat anti-rabbit antibody (Alexa Fluor 647, A31573, Invitrogen). The nuclei were stained with DAPI (268,298, MERCK). The images were made with a confocal microscope (Laser Scanning Confocal Microscope LSM710 ZEISS).

For immunohistochemistry staining, biopsies were fixed in paraformaldehyde, embedded in paraffin, and cut into 3 µm sections. After paraffin removal with xylol treatment, samples were heated to expose any masked antigens using a Real Target Retrieval Solution containing citrate buffer (pH 6.0, Dako). Samples were pre-treated with Real Peroxidase-Blocking Solution (Dako) to block the endogenous peroxidase. Tissue sections were stained with anti-von Willebrand factor (VWF) (ab6994, Abcam) and counterstained with nuclear hematoxylin. Positive staining was counted and expressed as the mean of 10 independent counts for each animal, quantified at 20x using the analysis program Image-J 1.53 g (National Institute of Health, United States).

The analyses of these staining were done completely blinded, associating a number to each sample that could not be related to the type of treatment.

### Rebaudioside A degradation and reabsorption analyses

Since Rebaudioside A is the most abundant compound (62.6%) present in the SG extract, a solution containing Rebaudioside A at 1% was analyzed before and after 40 min of its injection (2 ml) to two C57BL/6JRccHsd mice not submitted to any treatment. The analysis was carried out using HPLC equipment coupled to Q Exacmemtive hybrid quadrupole-orbitrap mass spectrometer (MS) from Thermo Scientific (Waltham, Massachusetts, United States) with electrospray ionization (ESI) interface in negative mode. For chromatographic separation, an omega Polar C18 column (100 mm × 2.1 mm, particle size 3 µm) from Phenomenex, Madrid, Spain. The mobile phases and separation gradient were used at a flow rate of 0.4 ml/min. Ultrapure water with 0.1% (v/v) of formic acid (solvent A) and ACN with 0.1% (v/v) of formic acid (solvent B), applying a gradient from 0 % to 100% B during 15 min and 100% B were maintained for 2 min and the post-run equilibration time was 5 min. The temperature of the columns was kept at 40°C and the injection volume was 20 µl. MS analyses were performed using the full scan mode of 200–3,000 m/z.

### Statistical analyses

All statistical analyses were carried out with GraphPad Prism 8 (GraphPad Software, La Jolla, CA). Comparisons between the groups were performed using the unpaired *t* test or the nonparametric Mann-Whitney U test. The means of the experimental groups were compared by using one-way ANOVA. *p* values < 0.05 were considered significant.

## Results

### Steviol glycosides solutions showed ultrafiltration and transport capacities *in vitro*

**FIGURE 1:**
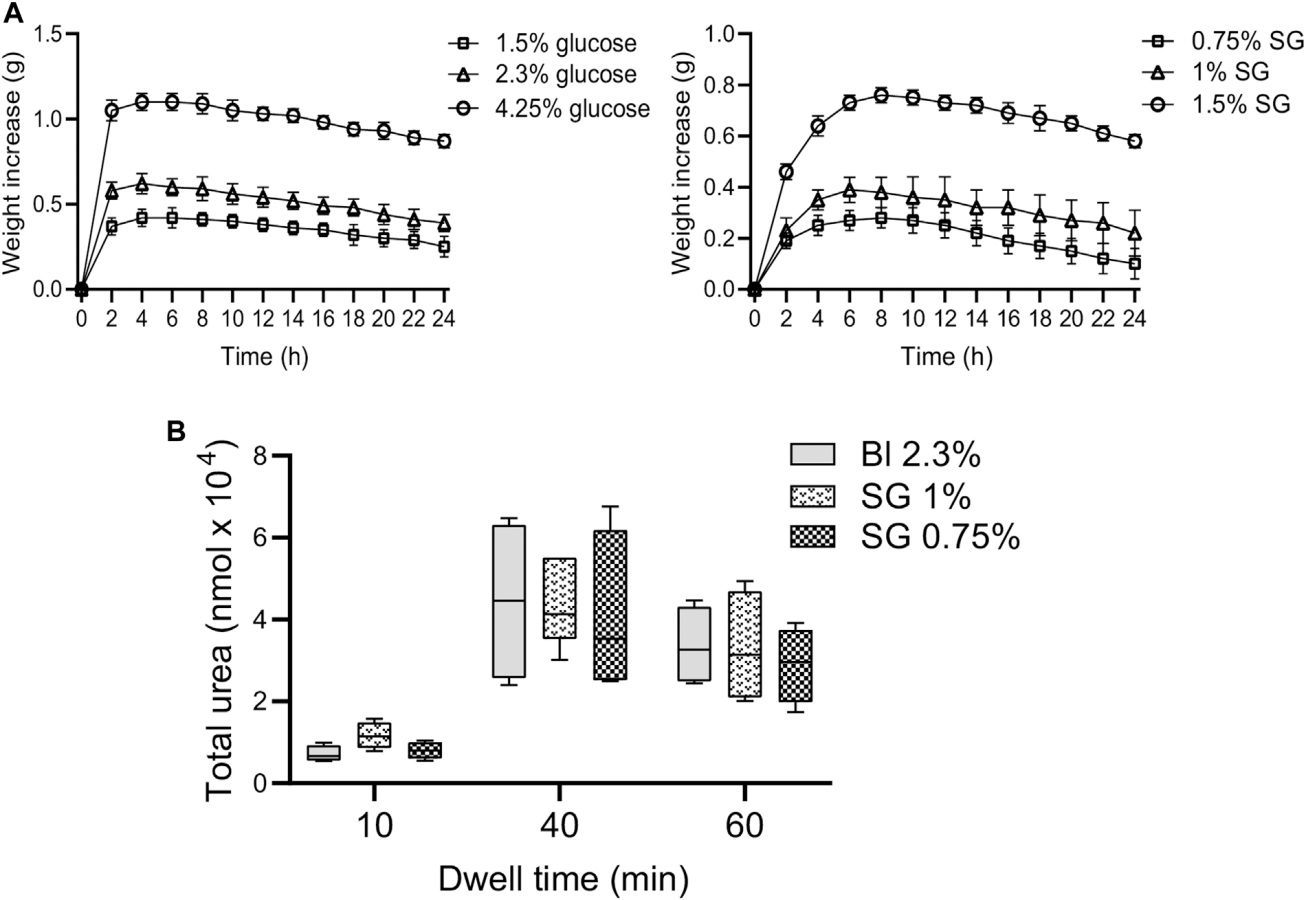
Trend curves of glucose and SG-based solutions and total urea measured in effluents. **(A)** Weight change curves of glucose 4.25%, 2.3%, and 1.5%, and SG solutions at 1.5%, 1%, and 0.75% measured at different time points using artificial membranes. Data are represented as mean ± SD of three independent experiments. **(B)** Total urea measured in drained effluents and extracted after 10, 40, and 60 min of dwell. Data are represented as median, maximum, minimum and the first and third quartiles. The analysis of variance results in a non-significant (NS) *p*-value (two-way ANOVA test).

The dialysis tubing bags containing different solutions were placed in a beaker filled with electrolytes (Na^+^, Mg^2+^, Ca^2+^, Cl^−^) in a lactate buffer and the mass change using artificial membranes was measured up to 24 h. Then, the weight increase (g) of each bag was calculated. SG-based fluids demonstrated a maintained and prolonged weight increase similar to glucose-based fluids, as indicated by the trend of their curves (Figure 1A).

Calculated crystalloid pressures exerted by SGs at the employed concentrations resulted isotonic to the blood, but the average MW of SG (905.50 Da) resulted in a value of 0.3183 for the reflection coefficient (*σ*), demonstrating that this compound has colloidal/oncotic osmotic capacity. (Supplementary Figure SC). Results *in vivo* indicated that SG 1% and 0.75% extracted urea from the blood to the peritoneal cavity in a similar-manner as Bl 2.3% after 10, 40, and 60 min (Figure 1B).

### Steviol glycosides has less impact on mesothelial cells alterations *in vitro*

**FIGURE 2:**
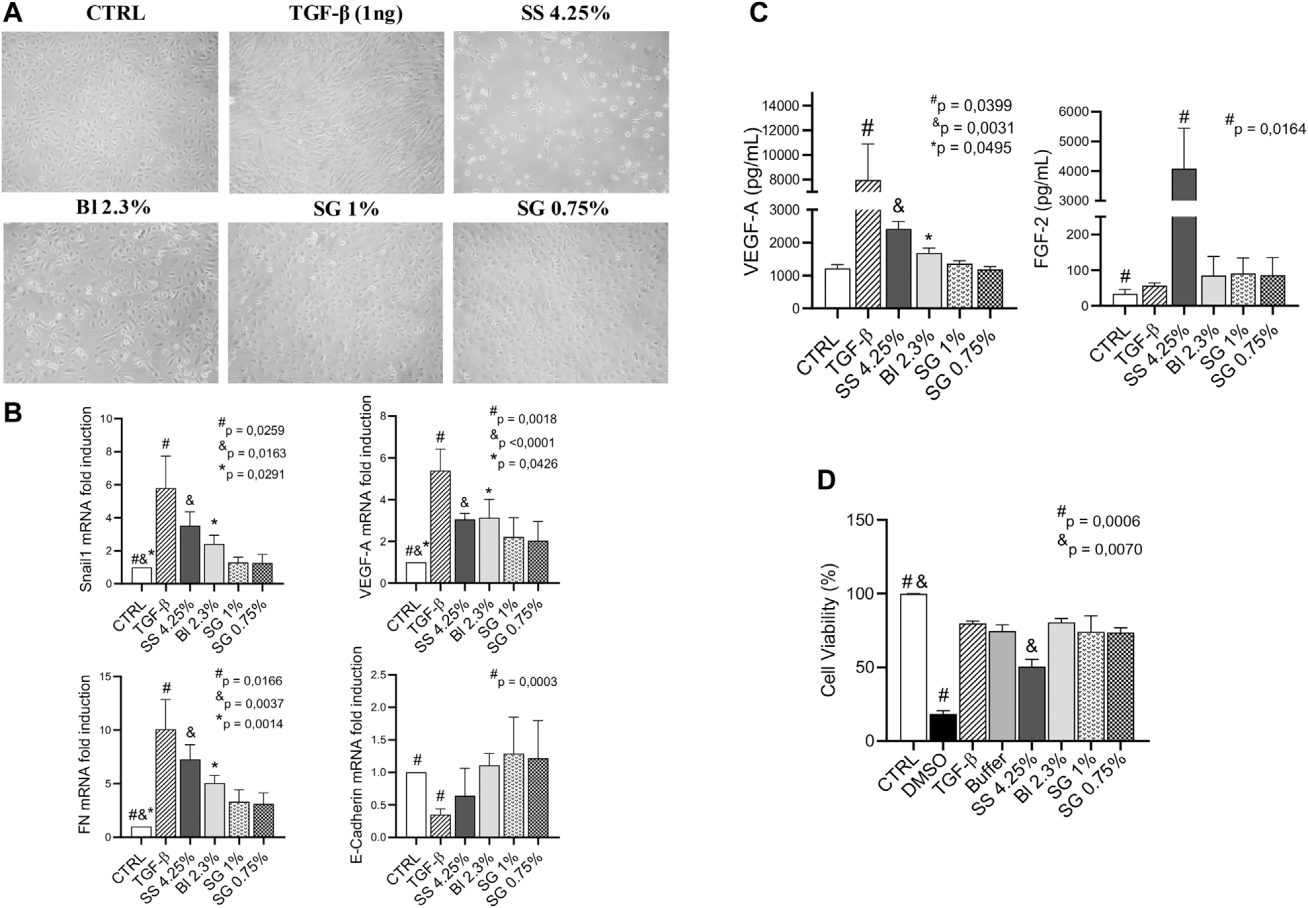
Effect of PDFs on HOMCs. **(A)** Effects of lactate buffer (CTRL), TGF-β1 (1 ng/ml), and different PD solutions (glucose and SG-based) on HOMCs morphology at 48 h. Pictures are representative of five independent experiments. **(B)** mRNA levels of different MMT markers analyzed by quantitative RT–qPCR. The results represent the relative expression of Snail1, VEGF-A, Fibronectin (FN), and E-Cadherin. The data are depicted as a mean ± SEM of five independent experiments. The analysis of variance results in a *p-*value = 0.0028; 0.0044; 0.0011 and NS respectively (one-way ANOVA test). **(C)** VEGF-A and FGF-2 concentration in supernatants analyzed by Luminex 100 (*p-*value = 0.0009 and 0.0154 respectively, one-way ANOVA). **(D)** Cellular viability of Met-5A incubated with DMSO 5%, TGF-β1 (1 ng/ml), lactate buffer, glucose, and SG-based solutions. The analysis of variance results in a *p-*value = 0.01 (one-way ANOVA test). Symbols show statistical differences between groups analyzed by the unpaired *t* test.

Sub-confluents HOMCs cultures were exposed for 48 h to glucose-based PDFs (SS 4.25%, as a positive control, or Bl 2.3%) and SG-based fluids (1% and 0.75%). The control group was incubated with a lactate buffer diluted one-half with a culture medium. MCs were lysed and RNA extracted for analysis by RT-qPCR. Exposure of HOMCs to SS 4.25% resulted in cell death and in changed morphology, becoming elongated after 48 h (Figure 2A). The morphology of HOMCs incubated with Bl 2.3% was slightly changed, which was confirmed by the measurement of levels of standard epithelial and mesenchymal markers by RT-qPCR. Glucose-based PDFs significantly upregulate the expression of Snail1, VEGF-A, and Fibronectin, whereas SG did not show regulation of these markers (Figure 2B). Angiogenic factors FGF-2 and VEGF-A were mainly affected by glucose treatment (Figure 2C). In addition, the expression of E-Cadherin was preserved with SG and Bl 2.3% fluids, while SS 4.25% showed a tendency to decrease this epithelial marker (Figure 2B). In agreement with these results, incubation of Met-5A cell line with Bl 2.3% or SG-based fluids did not have an effect on cellular viability, whereas incubation with SS 4.25 significantly reduced the cellular viability (Figure 2D).

### Steviol glycosides-based PD fluids did not induce significant mesothelial-mesenchymal transition, fibrosis, inflammatory response, or angiogenesis at the peritoneal tissue *in vivo*

**FIGURE 3:**
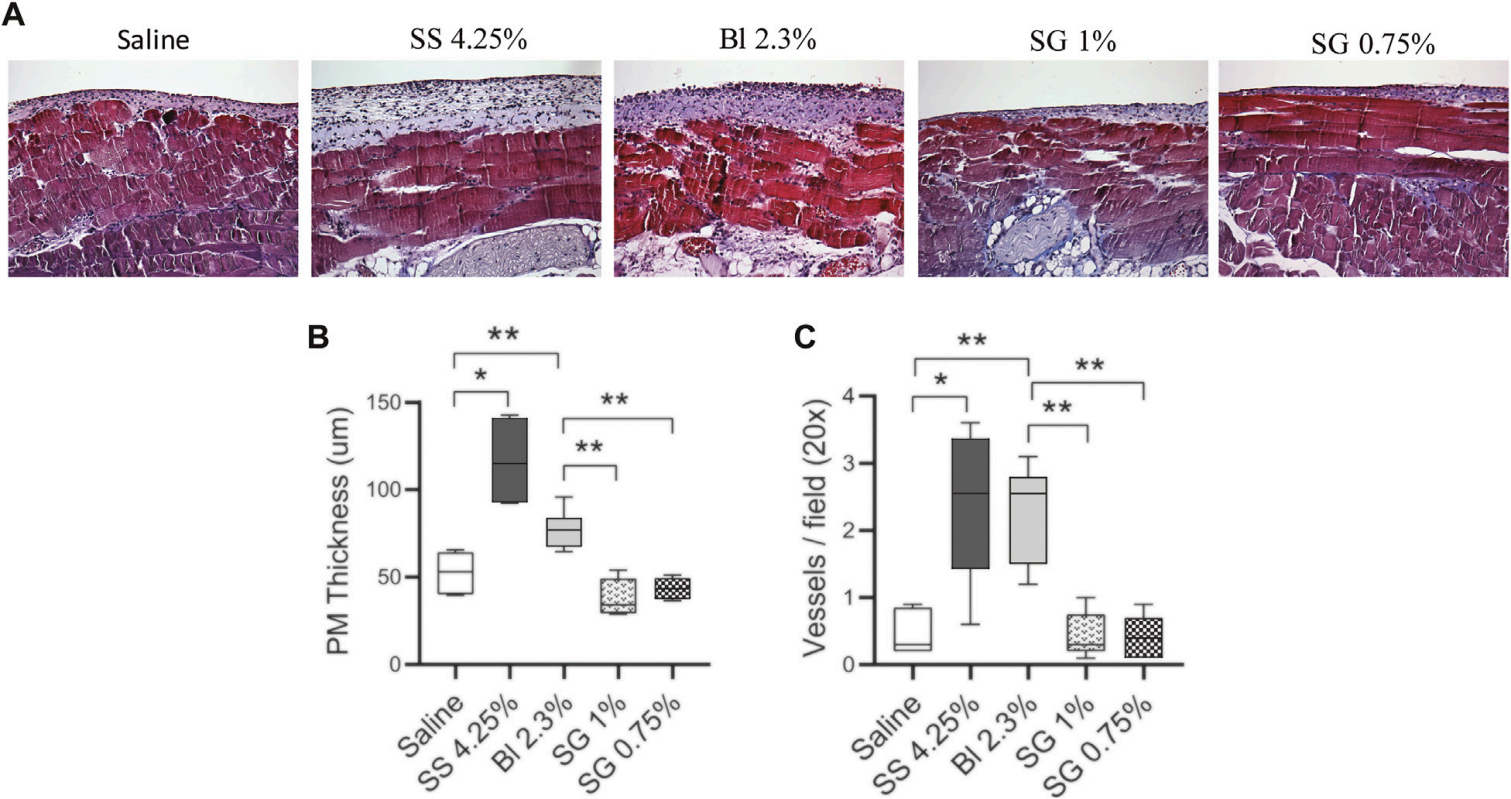
Peritoneal membrane alterations in mice. **(A)** Paraffin sections of the PM from all the groups were stained with Masson’s trichrome (20X). **(B)** The thickening of PM was determined by morphometric analysis. One-way ANOVA test results in a *p*-value *<* 0.0001. **(C)** Quantification of the total VWF positive cells in the PM (*p-*value < 0.0001, one-way ANOVA). Box plots graphics represent the median, minimum, and maximum values. Symbols represent the statistical differences between the groups analyzed by Mann-Whitney U test (*p < 0.05, **p < 0.01).

The mice were treated with glucose-based and SG-based fluids (2 ml twice per day) for 40 days and the thickness of the PM was evaluated. Masson’s trichrome staining of the peritoneal biopsies showed that SG groups had less submesothelial matrix deposition and cell infiltration than glucose-based PDFs (Figure 3A). Morphometric analysis showed that glucose-based PDFs had thicker PMs as compared with SG groups (Figure 3B).

In addition, the number of blood vessels in the submesothelial zone was counted. It was observed that the blood vessels increased in glucose-based PDFs and were not significantly affected in SG groups as compared with control mice (Figure 3C).

**FIGURE 4:**
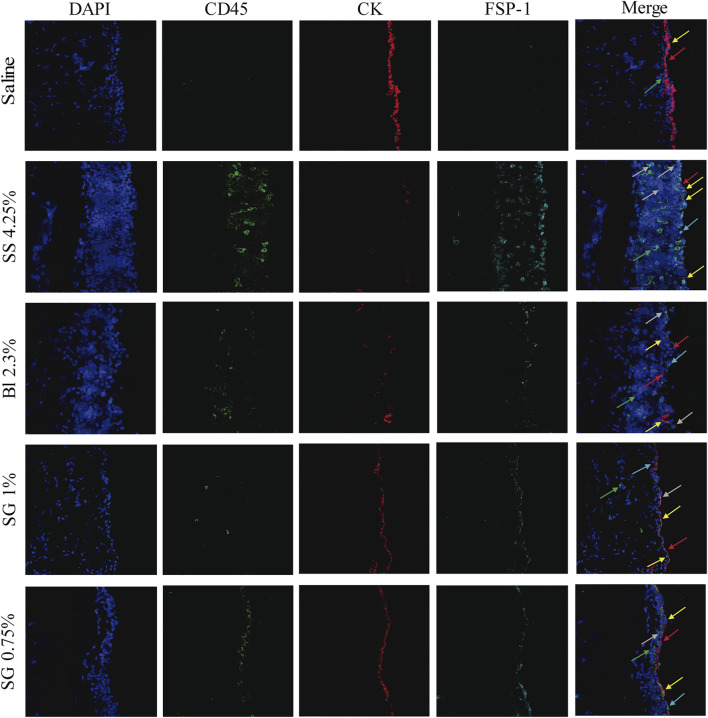
Immunofluorescence microscopy analysis of parietal peritoneal tissue sections. The presence of inflammatory (CD45, green), fibroblastoid cells (FSP-1, cyan), and mesothelial cells (Cytokeratin (CK), red) were determined in frozen sections of the PM. Yellow arrows show co-expression of FSP-1 and CK indicating MCs convert into myofibroblast by MMT process. Gray arrows show co-expression of FSP-1 with CD45^+^ indicating fibrocytes origin. The color balance was equally adjusted by using Image-J 1.53 g. The pictures are representative of each group.

Immunofluorescence analysis of peritoneal tissues showed that SS 4.25% induced high recruitment of CD45^+^ cells (green) in the submesothelial zone and that the MCs layer was not well preserved. Furthermore, it showed an accumulation of fibroblastoid cells expressing FSP-1 (cyan), some of them co-expressed cytokeratin (CK, yellow) that indicates MCs undergo the MMT process; some of them co-expressed CD45^+^ (gray arrow) showing myofibroblasts originated from bone-marrow-derived circulating cells (fibrocytes). In the Bl 2.3% group, the MC layer was better preserved and FSP-1^+^ and CD45^+^ cells were found in lower numbers than in SS 4.25%. The SG groups showed even better preservation of the mesothelium and FSP-1^+^ and CD45^+^ cells were less abundant (Figure 4).

### Steviol glycosides-based solutions induced a milder inflammatory response *in vivo*

**FIGURE 5:**
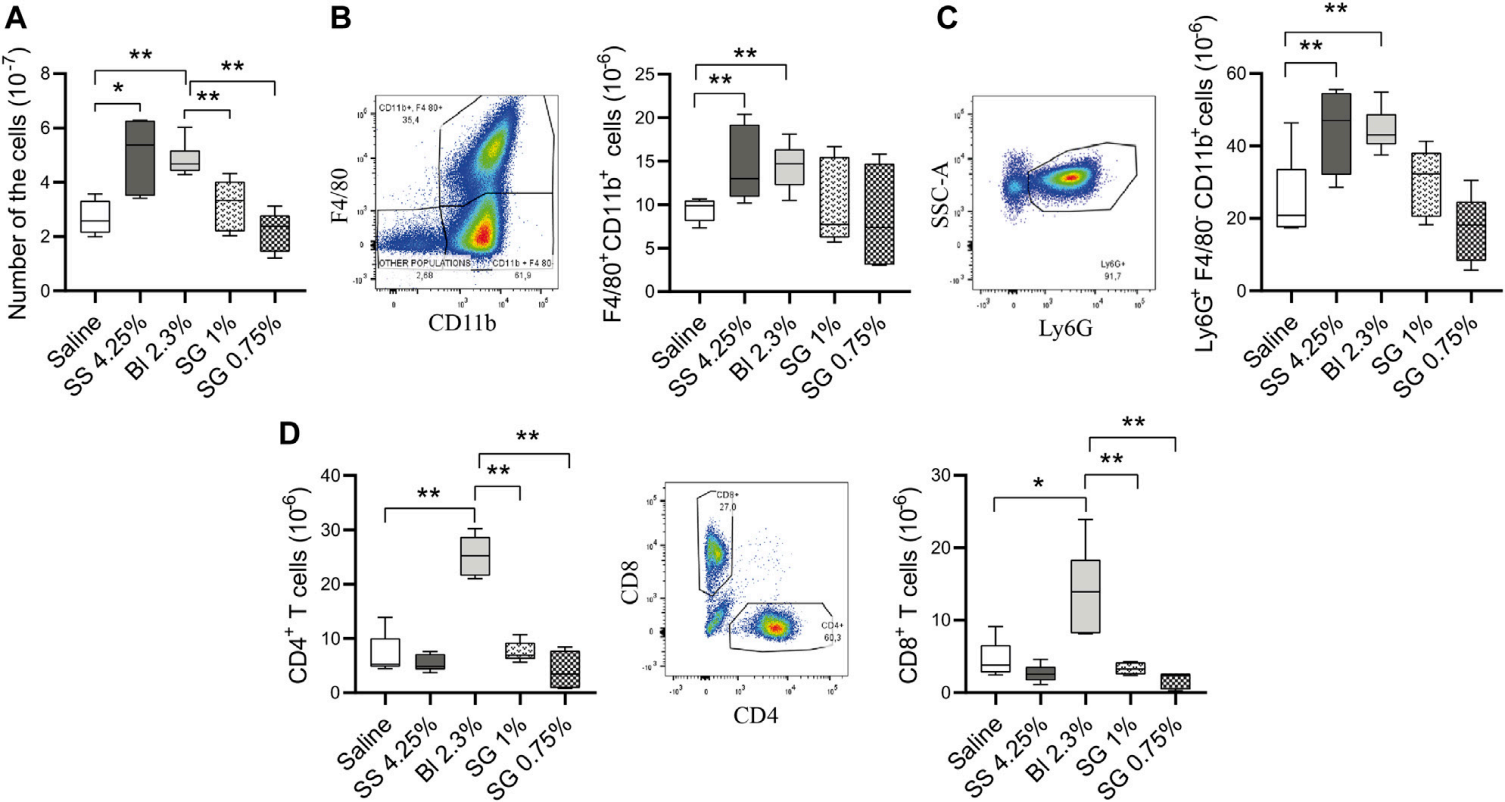
Cell populations and inflammatory status at the peritoneal washings. **(A)** Total numbers of nucleated cells. One-way ANOVA test results in a *p*-value of 0.0033. **(B)** Total numbers of macrophages (CD11b^+^F4/80^+^ cells) (*p* = NS, one-way ANOVA). **(C)** Total numbers of granulocytes (Ly6G^+^F4/80^−^CD11b^+^ cells). One-way ANOVA test results in a *p*-value of 0.0008. **(D)** Total numbers of CD3^+^CD4^+^ and CD3^+^CD8^+^ T cells (*p*-value < 0.0001, one-way ANOVA test). Box plots represent the median, minimum, and maximum values. Symbols represent the statistical differences between the groups analyzed by Mann-Whitney U test (*p < 0.05, **p < 0.01).

Thereafter, the levels of the infiltrating leukocytes were analyzed within the peritoneal cavity after the treatments. The total number of nucleated cells was not affected by SG solutions, while increased in glucose-based PDF in comparison with control (saline-treated) mice (Figure 5A). The level of macrophages was significantly increased in glucose-based groups, whereas the increment was less pronounced in SG groups, although they did not show a significant reduction when compared with Bl 2.3% (Figure 5B). Moreover, granulocytes, agents of the innate immune system, tended to increase in glucose-based PDF groups compared with saline and SG solutions (Figure 5C).

In addition, the presence of T cells was analyzed in the peritoneal cavity. The mice group treated with Bl 2.3% had a higher number of CD4^+^ and CD8^+^ T cells compared with control, SS 4.25%, and SG-treated mice (Figure 5D).

**FIGURE 6:**
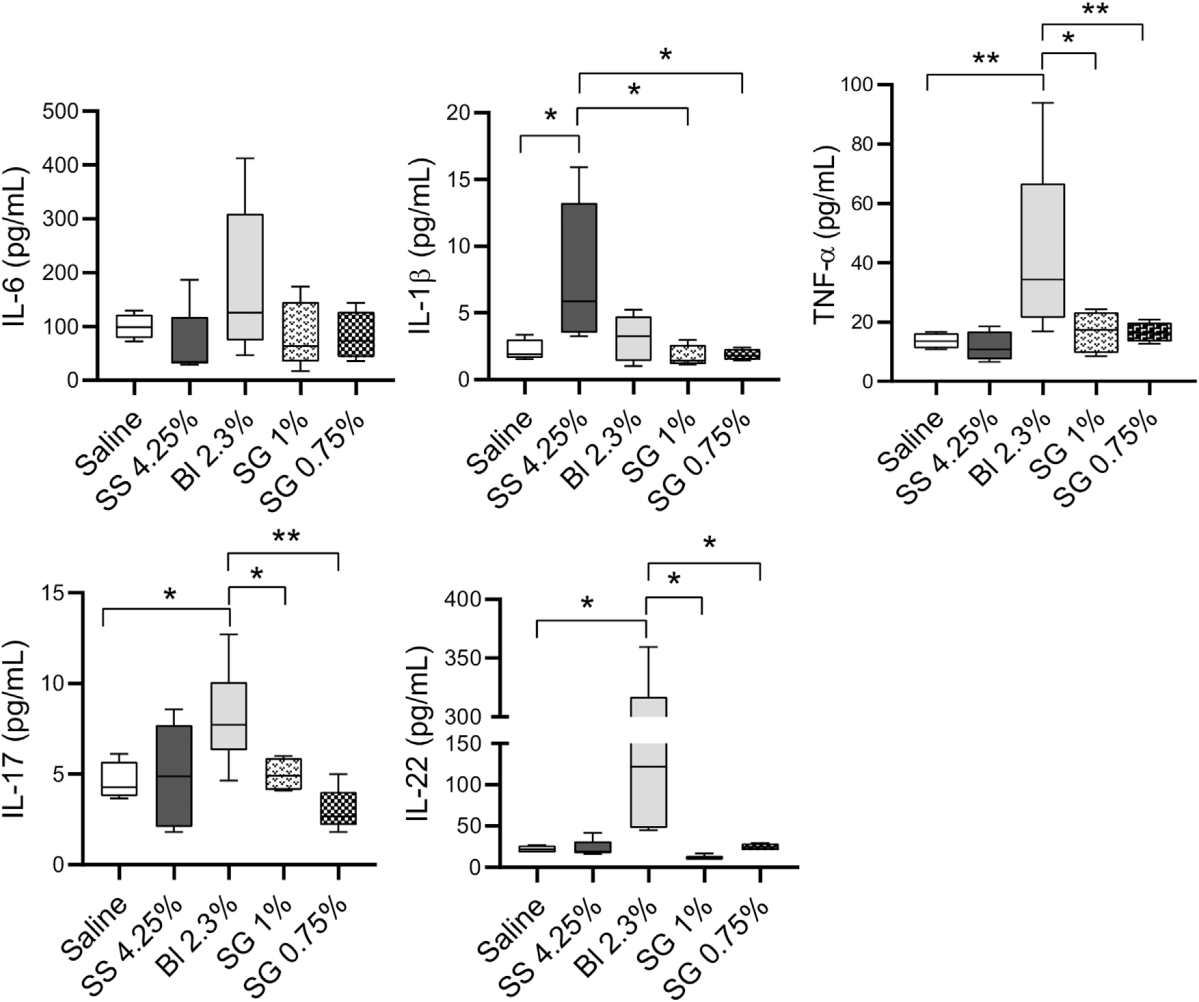
Cell populations and inflammatory status at the peritoneal washings. IL-6, IL-1β, TNF-α, IL-17, and IL-22 concentrations at peritoneal washings. One-way ANOVA test results in a *p*-value of 0.0042 for IL-1β, 0.0205 of TNF-α, 0.0059 for IL-17, and NS for IL-6 and IL-22. Box plots represent the median, minimum, and maximum values. Symbols represent the statistical differences between the groups analyzed by Mann-Whitney U test (*p < 0.05, **p < 0.01)

Regarding soluble factors analyzed in peritoneal washings, IL-6 concentrations do not show differences between groups, while there is an increase of IL-1β in SS 4.25% compared with the other groups, and of TNF-α, IL-17, and IL-22 in Bl 2.3% (Figure 6).

### Rebaudioside A did not degrade in the peritoneal cavity

The main components of the SG extract (Rebaudioside A and Stevioside) were preserved when compared with a pattern sample (data not shown). To explore the possible degradation of SG fluid i.p., its main compound was analyzed by HPLC equipment before and after 40 min dwell in mice. For that, its main compound (Rebaudioside A) was used to facilitate the measurement of degradation products. Rebaudioside A solution at 1% (weight/volume) was analyzed before being injected into mice, and we observed a purity of 96%, with a small amount of Rebaudioside B. Two mice were injected with this solution, and we observed that the proportion of both components remained equally constant after 40 min of dwelling. Therefore, no degradation was observed (data not shown).

## Discussion

Using glucose as an osmotic agent in PD has the advantage of being relatively safe and inexpensive, and is a source of calories. However, an increase in glucose absorption leads to worsening control of blood sugar (Daugirdas et al., 2015). The instillation of large amounts of glucose into the peritoneal cavity predisposes patients to hyperglycemia, dyslipidemia, obesity and cardiovascular alterations (Delarue and Maingourd, 2001; Balafa and Krediet, 2012). Long-term PM damage is caused, leastwise partially, by glucose or via GDP and the formation of AGEs, impairing UF (Witowski et al., 2003). The fact that severe peritoneal damage is still observed in patients infused with low-GDP PDFs solutions, suggests that glucose per se has a deteriorating effect on the PM. Moreover, glucose-based PDFs are not very effective in high transporters patients, and inadequate UF may result. High osmolarity itself is also a key factor affecting the biocompatibility of PDFs (Cendoroglo et al., 1998; Wong et al., 2003; Piccapane et al., 2020). Clinical studies have determined that patients who use large concentrations of glucose during the day are at the highest risk for developing a loss of UF capacity (Daugirdas et al., 2015). Continued use of the SS 4.25% PDF could theoretically result in the removal of 7.2–9.6 L per day and cause marked hypernatremia. In practice, this degree of fluid removal is rarely required (Daugirdas et al., 2015). Moreover, glucose-based PDFs are associated with reduced immunological defenses in the peritoneal cavity, since analyses on leukocytes demonstrate impairment of peritoneal immunocompetence, potentially contributing to an increased risk of PD-related peritonitis (Herzog et al., 2017). Therefore, the use of SS 4.25% glucose-based PDF, which contains high amounts of GDP, is decreasing nowadays worldwide at least for regular exchanges. For these reasons, we selected a glucose concentration of 2.3% (Bl 2.3%) to compare it with the candidate compound.

Regarding the ideal osmotic agent for PDFs, it has been determined that there is an optimal MW balancing the benefits of increasing molecular size (and the increased osmotic efficiency associated with this size increment) and the disadvantage of the increases in solute mass per molecule, (the increasing concentration in g/L). This seems to occur already at MW of around 1,000 Da (Rippe and CarlssOn, 1996). Thereby, given its therapeutic effects and its average MW (905 Da, while glucose is 180 Da), the SG mixture was considered a very interesting candidate for a PDF osmotic agent. In the case of high MW agents, colloid osmotic pressure is particularly relevant. Net ultrafiltration flux is the result of crystalloid and colloid/oncotic pressures (Devuyst and Rippe, 2014). The relationship between these pressures is determined by the Starling equation, which takes into account the reflection coefficient (*σ*) of the molecules. It indicates the probability of not being reabsorbed through three types of pores (large, small, and ultrasmall) (Rippe and CarlssOn, 1996) into the microvessel blood torrent and is an inherent property of the molecule given the charges, the size, and the topology. The estimated reflection coefficient (*σ*) of the SG mixture is ten times higher than glucose. It has been demonstrated with icodextrin (average MW = 5,000) that substances of about 4 nm are expected to be actively reflected by the pores to the peritoneal cavity (Rippe and Levin, 2000; Morelle et al., 2018). Icodextrin entails a mean hydrodynamic radius of 4 nm (1–23 nm), forming 25%–30% of colloidal fractions of highest MW (Morelle et al., 2018). Rebaudioside A, the major SG component, has self-nanomicellizing properties of up to nearly 4 nm (Hou et al., 2019; Song et al., 2020). Micelles are a type of colloid. Thereby, SG osmotic capacity is dependent on oncotic forces.

Using artificial membranes, we observed that 1.5%, 1%, and 0.75% SG-based fluids display prolonged and maintained ability to attract water *in vitro*. Checking *in vivo,* these concentrations (Bl 2.3%, 1% and 0.75% SG) were able to extract the same amount of urea after 10, 40, and 60 min in the peritoneal cavity. Based on the results, these concentrations were chosen for all further experiments.

The next step to determine SG benefits to be used for PDFs was to test the peritoneal status after SG exposure. MCs are the first line of contact of the body with PDFs, suffering alterations related to functional and structural peritoneal damages (Yáñez-Mó et al., 2003). To analyze their behavior in the presence of SG, HOMCs were exposed to the selected concentrations of SG for 48 h and their effects were compared with Bl 2.3% since longer exposition periods strongly affect the viability of cells treated with SS 4.25%. SG solutions did not affect morphology and ameliorate cellular viability in contrast with the deleterious effect of SS 4.25%. The morphology of MCs incubated with Bl 2.3% was slightly changed, as confirmed by standard mesenchymal markers measured by RT-qPCR. Glucose-based fluids induced mesenchymal markers (Snail1, VEGF-A, Fibronectin), while downregulated the epithelial marker (E-Cadherin) in the case of the highest concentration of glucose and GDP (SS 4.25%), which indicates the induction of an MMT process. This agrees with previous observations from our group in which we observed that low-GDP PDFs have less impact *in vitro* on MMT of MCs than a standard PDF (Bajo et al., 2011). None of the mentioned markers were significantly affected by SG treatment. An increasing number of studies indicate direct adverse effects of high glucose concentrations on cellular function. In line with Lee et al. (2004) we have also demonstrated the induction of the fibronectin expression in human peritoneal MCs upon exposure to high glucose in conventional PDF, thus favoring the pathogenesis of peritoneal fibrosis.

We have previously described that VEGF-A levels had marked differences between early (epithelioid phenotype) and advanced (non-epithelioid phenotype) stages of MMT, which in turn correlated with the elliptical factor values of effluent-derived MCs (Ruiz-Carpio et al., 2017). Here, we observed that VEGF-A levels measured by RT-qPCR and ELISA significantly increased when treating cells with glucose-based PDFs, but not with SG solutions. Since MMT plays a central role in the pathogenesis of peritoneal decline during PD (Yáñez-Mó et al., 2003), these results are very promising, as they demonstrate that SG is able to better preserve the epithelial morphology of MCs *in vitro* compared with glucose. In previous studies, it was suggested that FGF-2 and VEGF-A are the most potent angiogenesis inducers and have a synergistic effect on this process (Pepper et al., 1992; Asahara et al., 1995). Here we observed that angiogenic growth factor FGF-2 was mainly affected by SS 4.25% treatment. Previously, it was demonstrated that exposure to high glucose levels caused a concentration-dependent increase of FGF-2 mRNA expression and secretion by HOMCs, indicating that MCs are one of the peritoneal sources of this factor (Ogata et al., 2001).

To go further to unravel the biological effects of SG, we analyzed them *in vivo* in a mice model of peritoneal PDF exposition. It has been previously described that glucose-based PDFs induce fibrosis, angiogenesis, MMT, and leukocyte infiltration within the peritoneum (González-Mateo et al., 2014; Liappas et al., 2016). All these biomarkers are clearly reduced when administering SG solutions in comparison with glucose-based PDFs, which demonstrates that SG is a much more biocompatible solution.

During the peritoneal thickening process, there is a progressive accumulation of myofibroblasts. Here, we show that the accumulation of myofibroblasts is greater in the peritoneal samples from glucose-based PDFs exposed animals than in SG-based PDF treatments. Concretely, in the SS 4.25% group, there is a remarkable infiltration of CD45^+^ cells, and many of these cells co-express FSP-1, a fibroblastoid cell marker, suggesting that these cells are fibrocytes generated from progenitors of hematopoietic origin that are recruited to the injured tissues (Loureiro et al., 2011). Besides this, SG maintains the integrity of the mesothelial layer, while glucose disrupts it in a concentration-dependent manner. Although the mesothelial layer deteriorates in the SS 4.25% group and most MCs are lost, a significant proportion of the ones that remain in the tissue express FSP-1, indicating that these fibroblastic cells derive from mesothelial cells via MMT (Loureiro et al., 2011).

Moreover, intact mesothelium provides *in vivo* resistance against solute permeation. Damage of intercellular junctions leads to an increase in solute permeability. High glucose concentration, along with high osmolarity, has been probed to damage intercellular junctions in HOMCs (Ito et al., 2000; Ito and Yorioka, 2008). The high paracellular permeability is a characteristic of the so-called “high transporters” PD patients (Correa-Rotter and Cueto-Manzano, 2001), in which the osmotic gradient between the PDF and the blood is rapidly dissipated, leading to poor UF. Taking this into account, SG-based PDF, while preserving the integrity of the mesothelial layer as observed *in vitro* and *in vivo*, and given that its bigger MW reduces the diffusion speed to the blood of the compound, would guarantee a better UF capacity.

Another fact directly implicated in UF capacity is the number of capillaries. Angiogenesis is an important component of PM failure and it has been associated with alterations in the peritoneal water transport (Krediet et al., 2000). In this study, an increase in the number of vessels per field is observed in the glucose-based PDFs groups compared with control, whereas in SG-based fluids such increase is not produced. This data agrees with the induction of the pro-angiogenic factors VEGF-A and FGF-2 by HOMCs treated with glucose-based PDFs in contrast with cells treated with SG-based fluids, previously observed *in vitro*. This fact indicates again that SG would better maintain UF capacity.

Peritoneal effluent is a source of inflammatory cells recruited to the peritoneal cavity and of biomarkers produced by both, these cells and the ones present in the tissue. Inflammatory processes are key in modulating wound healing, angiogenesis, and fibrosis. It is known that macrophages play an important role in the fibrotic process through the secretion of growth factors. T cells are responsible for the orchestration of inflammatory responses through the secretion of cytokines that play a role in fibrosis by the activation of fibroblasts (Theiss et al., 2005; Doucet et al., 2008) and recruitment of fibrocytes (Shao et al., 2008). Here we observed that peritoneal exposure to glucose-based PDF tended to increase cell numbers in the peritoneal cavity, mainly macrophages (CD11b^+^F4/80^+^ cells). To a lesser extent, there is also a tendency to increase the number of granulocytes (CD11b^+^F4/80^-^Ly6G^+^ cells), while SG-based fluids maintained a low number of these cells, similar to the control group.

The numbers of CD4^+^ and CD8^+^ T cells increased as well in the Bl 2.3% group as observed before (Aroeira et al., 2009; González-Mateo et al., 2014). Bl 2.3% demonstrated to better maintain HOMCs viability compared with SS 4.25%. Therefore, it could additionally preserve the viability of other cell types and thus exert better immunocompetence than SS 4.25%. It has been suggested that CD4^+^ T cells are the primary source of IL-17 in the peritoneum of mice treated with PDF and that this cytokine plays an important role in the generation of PDF-induced peritoneal fibrosis in patients and mice exposed to PDF (Rodrigues-Díez et al., 2014). Here there is an increase of IL-17 in the Bl 2.3% group, but not in the others.

In the case of IL-22, is produced by several populations of immune cells at the site of inflammation. Producers are *αβ* T cells classes Th1, Th22, and Th17 along with γδ T cells, NKT, ILC3, neutrophil granulocytes, and macrophages (Dudakov et al., 2015). As commented above, T cells and macrophages do significantly increase and total granulocytes tend to enhance in the Bl 2.3% injected mice. Here, IL-22 concentrations in peritoneal washings increase in the Bl 2.3% group compared with the control. IL-22 exerts its effect on non-hematopoietic cells, mainly stromal and epithelial cells, stimulating cell survival and participating in wound healing, and proliferation and synthesis of antimicrobials (Dudakov et al., 2015). It has been described that IL-22 might protect from fibrosis (Gu et al., 2021) and MMT of MCs (Ye et al., 2015). Thus, we hypothesized that this cytokine protects the Bl 2.3% treated group from MMT and fibrosis, counteracting the effect of IL-17.

Moreover, *Stevia* extracts have been proven to downregulate inflammatory-related response markers such as IL-1β and TNF-α (Mehmood et al., 2020; Mostafa et al., 2020). Here we have observed an increase of IL-1β and TNF-α in washings of mice treated with SS 4.25% and Bl 2.3% PDFs respectively, while it is not observed in SG-based fluids-treated mice. Regarding other cytokine production, we have previously observed that IL-6 could slightly increase during long-term PDF exposure, without reaching statistical significance (González-Mateo et al., 2014; Liappas et al., 2016). Here, we also observed no differences among groups in peritoneal washings concentrations.

In addition, it has been described that Rebaudioside A and Stevioside do not undergo browning or caramelization when heated (Joint Expert Committee on Food Additives, 2005). Considering its safety to be used *in vivo*, we evaluated the absorption of SG into the body and its degradation to discard toxicity. It has been shown that the mean absorption rate when using 4.25% and 2.3% of glucose dwelled for 4 h was 64.4% in humans, ranging from 30.6% to 92.4%, (Zuo et al., 2015). Another study revealed absorption of 75% of the initial glucose concentration at the end of a 6 h dwell. The percentage of glucose absorbed overtime was almost identical for the 3 different concentrations used in the clinical practice (1.5%, 2.3%, or 4.25%) (Heimbürger et al., 1992). Studying in isolation Rebaudioside A (with a 3.6% of Rebaudioside B), the majority component of the SGs mixture, we observed absorption of 40%–60% in 40 min in mice exposed to 1% concentration. This component has self-nanomicellizing properties, so it **can** be hypothesized that a percentage of it will form aggregates of bigger size (Hou et al., 2019), leading to a slow absorption rate. Most important is that no degradation was observed, since no other compounds were detected in effluents and the percentages of the Rebaudioside A and B remain invariables, indicating that there is no generation of any toxic metabolic product. Further analyses regarding the possible accumulation of these compounds in the body and their implications should be done in the future.

In this regard, it has been reported that, after oral administration, SG are not modified during their way through the digestive system until the colon. In the colon the Stevioside and the Rebaudioside A are hydrolyzed to Steviol (318.45 Da), although the hydrolysis of Rebaudioside A is slower than that of Stevioside. It has been confirmed that Rebaudioside A showed stability when exposed to *in vitro* matrices simulating stomach and small intestine fluids, with susceptibility to hydrolytic degradation by enteric bacteria collected from the cecum (Nikiforov et al., 2013). Incubations with rat liver microsomes indicated that it is not expected to be metabolized by the liver enzymes. Plasma concentrations of Rebaudioside A and/or its final hydrolysis product, free/conjugated Steviol, were consistent between animals administered Rebaudioside A in the diet. In humans treated orally with Stevioside, small amounts of Steviol were detected in the plasma, with considerable interindividual variability. A dietary toxicity study (repeated exposure 2000 mg/kg/d Rebaudioside A) observed that there were no treatment-related effects on the general condition and behavior of the animals and no toxicologically relevant, treatment-related effects on hematology, serum chemistry, or urinalysis. Macroscopic and microscopic findings revealed no treatment-related effects on any organ evaluated (Nikiforov et al., 2013). Together with our results, these findings confirm that this compound seems to be free of toxicity at tested concentrations.

Stevioside and/or Steviol affected a variety of biochemical parameters in *in vitro* models, indicating possible anti-hypertensive and anti-glycemic effects. Stevioside and Rebaudioside A have not shown evidence of genotoxicity *in vitro* or *in vivo*. (Joint Expert Committee on Food Additives, 2005).

Novel osmotic agents have also been recently proposed as substitutes for glucose in PDFs, such as xylitol and L-carnitine (Bazzato et al., 1982; Rago et al., 2021). These compounds showed as well interesting properties, and their behavior corresponds to crystalloid osmosis since they have a MW similar to glucose.

Recent studies about glucose sparing (Piccapane et al., 2020; Masola et al., 2021) suggested a combination of two or three osmotic agents replacing a substantial part of glucose to improve the PD solution biocompatibility. The authors maintained some glucose in the PD solution affirming that it is still a key nutrient for patients affected by malnutrition. The suggestion of mixing two or even more osmotic agents for PDFs is not novel, as it was already proposed more than 40 years ago (Khanna et al., 1986). In this regard, to achieve higher osmotic capacities for special requirements, it could be interesting to combine SG and glucose, so that none of the two osmotic agents needs to be present at high concentrations.

A large body of evidence tends to show that *stevia* and SG are safe for human consumption at the dietary level. Nevertheless, their clinical efficacy and safety for PD still require further pre-clinical studies. Therefore, the results of the present report are preliminary and the usefulness of SG as an osmotic agent advocates for more studies to provide in-depth insights into its safety, health benefits, and physiological mechanisms. In any case, this study opens a new door toward a possible new substitute for glucose as an osmotic agent. In conclusion, SG has demonstrated good qualities to be used as an osmotic agent for PDF, with fewer side effects compared with glucose.

## Data Availability

The raw data supporting the conclusions of this article will be made available by the authors, without undue reservation.
