# Supplementary figures and images for: Steviol glycosides as an alternative osmotic agent for peritoneal dialysis fluid
Source: Front Pharmacol. 2022 Aug 16;13:868374. doi: 10.3389/fphar.2022.868374 (PMC9424724; doi:10.3389/fphar.2022.868374)

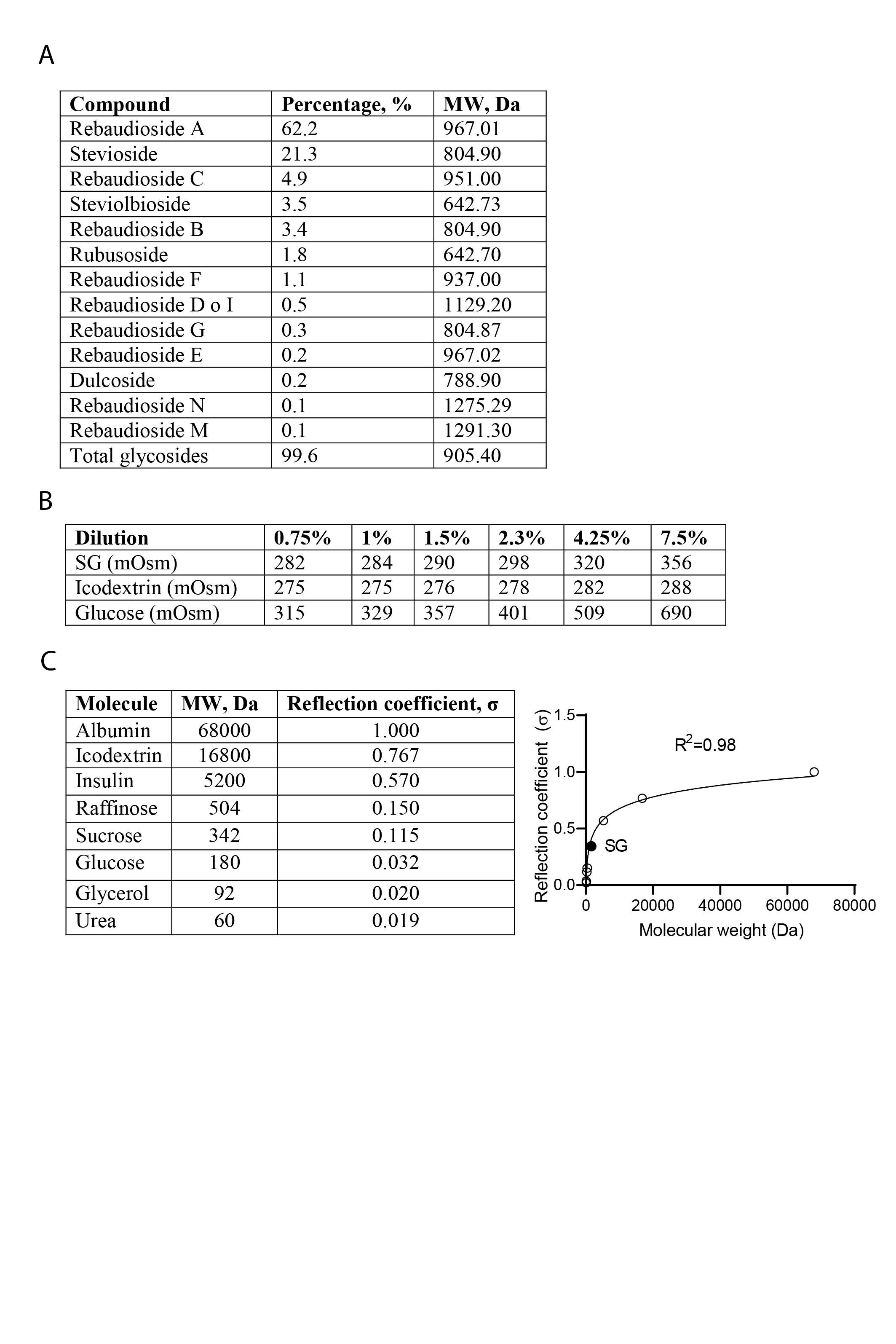

Supplement: Supplementary file 1 [file Image1.tif]
